# Safe Practices in Intravenous Drug Therapy in Hospitalized Adult Patients: Scoping Review

**DOI:** 10.1590/0034-7167-2025-0384

**Published:** 2026-09-28

**Authors:** Hevelyn dos Santos da Rocha, Milena Cristina Couto Guedes, Maithê de Carvalho e Lemos Goulart, Fernanda Garcia Bezerra Góes, Asaphe Stenio Lima dos Santos, Fernanda Maria Vieira Pereira-Ávila

**Affiliations:** I Universidade Federal Fluminense. Rio das Ostras, Rio de Janeiro, Brazil

**Keywords:** Infusions, Intravenous, Nursing Care, Nursing, Hospitals, Adult., Infusiones Intravenosas, Atención de Enfermería, Enfermería, Hospitales, Adulto.

## Abstract

**Objectives:**

to map safe practices in intravenous drug therapy in hospitalized adult patients described in the scientific literature.

**Methods:**

Scoping review according to JBI, with the question: “What are the practices for safe administration of intravenous drug therapy in hospitalized adults?”. Searches were conducted in four information resources and grey literature. Duplicates were removed in Endnote and studies were selected in Rayyan, according to eligibility criteria. The data were presented according to the Preferred Reporting Items for Systematic Reviews and Meta-analyses Extension for Scoping Review.

**Results:**

Eighteen studies were analyzed, and the evidence was grouped into the following stages: 1) Aseptic techniques; 2) Preparation; 3) Labeling; 4) Administration; 5) Recording. The following were highlighted as safe practices: hand hygiene, double checking, identification of medications and patients, verification of access permeability, recording of administration, and adverse events.

**Conclusions:**

adopting safe practices during intravenous drug therapy is a fundamental strategy for the safety of hospitalized adult patients.

## RESUMEN

**Objetivos:**

mapear las prácticas seguras en la terapia farmacológica intravenosa en pacientes adultos hospitalizados descriptas en la literatura científica.

**Métodos:**

se trata de una revisión de alcance según JBI, con la pregunta: “¿Cuáles son las prácticas seguras para administrar la terapia farmacológica intravenosa en adultos hospitalizados?”. Se efectuaron búsquedas en cuatro recursos informativos y en la literatura gris. Se eliminaron los duplicados en *EndNote* y se seleccionaron los estudios en *Rayyan*, respetando los criterios de elegibilidad. Los datos se presentaron según el PRISMA-ScR.

**Resultados:**

se analizaron 18 estudios cuyas evidencias se agruparon en las siguientes etapas: 1) Técnicas asépticas; 2) Preparación; 3) Etiquetado; 4) Administración; 5) Registro. Se destacaron como prácticas seguras: higiene de las manos, doble comprobación, identificación de los medicamentos y del paciente, verificación de la permeabilidad del acceso, registro de la administración y eventos adversos.

**Conclusiones:**

la adopción de prácticas seguras en las etapas de la terapia farmacológica intravenosa es fundamental para la seguridad del paciente adulto hospitalizado.

## INTRODUCTION

Patient safety is defined by the World Health Organization (WHO) as the absence of preventable harm and the reduction of unnecessary harm associated with healthcare^(1)^. In this context, medication errors directly affect patient safety and are characterized as any preventable event that may cause or result in the inappropriate use of medications, as well as harm to the patient, during the period in which the therapy is under the responsibility of the healthcare professional, patient, or consumer. Such errors may be linked to professional practice, the product, procedure, and health systems, ranging from prescription to monitoring and use of medications^(2)^.

According to the WHO, unsafe medication practices and medication errors are one of the main factors causing harm to patients, affecting the safety of 1 in 30 patients, most of which is preventable. In addition, it is estimated that the costs associated with these errors worldwide amount to $42 million annually. As a result, in 2017, the WHO defined medication without harm as the third global challenge for patient safety^(1,3)^. In the international context, medication errors cause 1.3 million injuries annually^(4)^, 9% of medication errors in hospitals occur during the medication administration stage^(5)^ and approximately 25% of the events found in medical records are related to drug therapy^(6)^.

The Brazilian scenario is no different, with around 30% of doses administered in hospitals in different regions of the country containing some type of error, with 1,500 errors related to medication administration in clinical inpatient units^(2)^. Errors occur predominantly during the preparation and administration of medication and may be associated with several factors, such as overburdened hospital units, professional fatigue, inadequate environmental conditions, and insufficient knowledge^(5,7-9)^.

The consequences of medication errors can be exacerbated depending on several factors, including the patient’s age. It should be noted that adults between the ages of 18 and 59 are in the process of aging and are therefore more susceptible to drug interactions and harm. An integrative literature review that analyzed 22 articles between 2018 and 2023 on patient safety and medication errors in the same population group identified that the main errors also occur in preparation and administration, with emphasis on omission or dosage errors, preparation failures, and inadequate labeling^(10,11)^.

These issues can be even more serious when administering parenteral medications, which have a systemic effect, such as intravenous drug therapy, which involves the infusion of fluids and medications through an intravenous (IV) line. In this case, adverse events resulting from errors are often associated with clinical symptoms and bloodstream infections (BSIs) caused by the infusion of contaminated solutions^(5,12)^, in the patient’s vascular access, which can be peripheral or central. It is estimated that 80% of hospitalized patients use these devices, which increases the risk of adverse events^(13,14)^.

In IV drug therapy, although the multidisciplinary team is involved in the process of preventing medication errors and promoting patient safety, the nursing team plays an extremely important role. Considering that nursing professionals are present throughout the process and are the last barrier to prevent possible harm to the patient, it is essential to ensure the safe preparation, administration, and monitoring of drug therapy^(7,15,16)^.

Furthermore, it is necessary to implement strategies and processes, in addition to strengthening the culture of patient safety^(16)^, to this end, it is essential that nurses master pharmacokinetics, pharmacovigilance, and all stages of IV drug therapy, considering its specificities and risks to adult patients^(17,18)^. Intravenous drug therapy is widely used in hospitals, especially in the care of adult patients, and is essential for the treatment of various clinical conditions. However, despite its recognized importance in healthcare practice, gaps remain regarding the lack of standardization in the processes involved, which compromises the uniformity of practices from the preparation and administration of drugs to the monitoring of therapeutic effects and the identification of possible adverse reactions.

The mapping of safe practices in IV drug therapy is therefore essential in order to collaborate with evidence-based practice and contribute to informed clinical decision-making based on current evidence and national and international guidelines on the subject. In addition, the evidence available in the literature is scattered and addresses practices in a fragmented manner, often restricted to isolated stages of the therapeutic process or specific contexts of practice. In this sense, a scoping review is relevant because it allows for the synthesis and integration of this information, bringing together, in a single study, the set of safe practices related to intravenous drug therapy in hospitalized adult patients.

Thus, this scoping review can contribute to improving professional practice, reinforcing the role of nursing staff in preventing medication errors and promoting patient safety, as well as contributing to the development and/or updating of clinical protocols, guiding professional training programs, and encouraging new research in the area, thereby contributing to the strengthening of a culture of patient safety. A preliminary search was conducted on April 25, 2025, in Prospero Systematic Review and Open Science Framework (OSF), but no other current or ongoing reviews or review protocols on the topic were identified. It should be noted that there are primary studies on the topic, ensuring the feasibility of this scoping review.

## OBJECTIVES

To map safe practices in intravenous drug therapy in hospitalized adult patients described in the scientific literature.

## METHODS

### Protocol and Registration

This is a scoping review prepared in accordance with JBI guidelines^(19)^, with the aim of ensuring transparency and quality, and followed the recommendations contained in Preferred Reporting Items for Systematic Reviews and Meta-Analyses extension for Scoping Reviews (PRISMA-ScR)^(20)^. The steps proposed by the method were followed, which consisted of: defining and aligning the research objective; establishing inclusion criteria with the objective and research question; describing the planned approach for searching, selecting, and extracting data; conducting the search, selection, extraction, and analysis of evidence; presenting the results obtained; synthesizing the evidence in relation to the objective of the scoping review, drawing conclusions, and highlighting the implications of the findings. The scoping review was duly registered on the OSF platform, DOI: 10.17605/OSF.IO/HV7G2.

### Review question

To construct the research question, a methodological strategy based on the acronym PCC was used - P (Population), C (Concept), and C (Context). In which P (population): adults; C (concept): safe practices related to intravenous drug therapy; C (context): adult patients who are hospitalized. Based on these elements, the following research question was formulated: “What are the practices for the safe administration of intravenous drug therapy in hospitalized adult patients?”.

### Eligibility criteria

For the consideration of the population, concept, and context, studies addressing safe practices and errors related to the administration of intravenous drug therapy in adults in a hospital setting were included. Studies addressing errors were considered, given that the occurrence of these events contributes to guiding safe practice actions in the healthcare environment.

The study adopted as an inclusion criterion the time frame of the last 10 years (2015-2025). This time restriction was established to ensure the inclusion of recent studies that reflect current practices, guidelines, and scientific advances in the field. The location or language of the studies was not considered.

Duplicate articles were excluded and counted only once. In addition, studies that presented specific guidelines for the safe and effective management of medications that are most frequently used in specific sectors of the hospital environment, such as vasoactive drugs used in the intensive care unit, were excluded.

### Sources of information

Designs from experimental and quasi-experimental studies, clinical trials (randomized or not), time series, epidemiological, observational, cross-sectional, analytical, prospective or retrospective cohort studies, case series, individual case reports, and relevant grey literature, such as clinical guidelines, manuals, and organizational documents, were considered. It also included qualitative research and systematic reviews. Studies such as letters, comments, editorials, expert opinion articles, and event summaries were excluded. We expect to receive a variety of materials and evidence to help us define the scope of the topic.

### Search Strategy

The searches were conducted in the following information resources: Medical Literature Analysis and Retrieval System Online (Medline) via PubMed, Virtual Health Library, Web of Science, and Scopus, accessed through the Coordination for the Improvement of Higher Education Personnel (CAPES) portal. In addition, to cover the grey literature, documents from health authorities such as the World Health Organization (WHO), Centers for Disease Control and Prevention (CDC), Ministry of Health (MS), and National Health Surveillance Agency (ANVISA) available in their virtual libraries were considered.

The initial search strategy was developed with the help of a librarian, and the search and selection of studies were carried out in three distinct stages: 1- Identification of keywords in titles and abstracts; 2- Use of keywords to search a database, which was carried out in the PubMed database; 3- Searching the established databases and applying the eligibility criteria. Steps one and two contributed to the construction of the search strategy/expression to be used in the third stage. Initially, words present in titles and abstracts were analyzed, as well as subject terms describing the articles. Subsequently, the keywords and subject terms identified were used in the complete database search. Finally, the search was conducted using descriptors from the controlled vocabularies in English, from the Medical Subject Headings Section (MeSH) with Boolean operators AND and OR, associating keywords and their variations in the title, abstract, and subject fields of the document. The searches were performed on May 8, 2025 (Chart 1).

**Chart 1:** Recording of search strategies for retrieving publications in databases, Rio das Ostras, Rio de Janeiro, Brazil, 2025

Search Registry			
**Source of Information**	**Search Strategy**	**Result** **No filter**	**Result** **With 10-year filter**
MEDLINE/ PubMed	((Adult) AND (“Infusions, Intravenous”[mesh] OR “Intravenous Infusions” OR “Infusion, Intravenous” OR “Intravenous Infusion” OR “Intravenous Drip” OR “Drip, Intravenous” OR “Drip Infusions” OR “Drip Infusion” OR “Infusion, Drip” OR “Infusions, Drip”)) AND (Hospitals)	18.603	4.757
BVS	(adult) AND (“Infusions, Intravenous” OR “Intravenous Infusions” OR “Infusion, Intravenous” OR “Intravenous Infusion” OR “Intravenous Drip” OR “Drip, Intravenous” OR “Drip Infusions” OR “Drip Infusion” OR “Infusion, Drip” OR “Infusions, Drip”) AND (hospitals)	832	276
Scopus	TITLE-ABS-KEY (((adult) AND (“Infusions, Intravenous” OR “Intravenous Infusions” OR “Infusion, Intravenous” OR “Intravenous Infusion” OR “Intravenous Drip” OR “Drip, Intravenous” OR “Drip Infusions” OR “Drip Infusion” OR “Infusion,Drip” OR “Infusions, Drip”) AND (hospitals)))	4.592	1.727
Web of Science	((ALL=(Adult)) AND ALL=(“Infusions, Intravenous” OR “Intravenous Infusions” OR “Infusion, Intravenous” OR “Intravenous Infusion” OR “Intravenous Drip” OR “Drip, Intravenous” OR “Drip Infusions” OR “Drip Infusion” OR “Infusion, Drip” OR “Infusions, Drip”)) AND ALL=(Hospitals)	293	136

### Selection of evidence sources

The search results were initially exported to Endnote Web reference management software, where duplicates were removed, and then exported to Rayyan software, used to organize and conduct the selection process for the studies that would comprise the scoping review. Both software programs are available online and free of charge.

The selection of evidence was conducted by two previously trained reviewers who analyzed the articles individually, with peer blinding, on separate devices, without interference or knowledge of the other reviewer’s selection. In case of disagreement between the two reviewers in the selection process, the disagreements were resolved by consensus between the two reviewers.

Initially, the titles and abstracts were read by pairs of independent reviewers to identify studies that met the eligibility criteria. The selected studies were read in full to exclude those that did not answer the research question of this review.

### Data extraction

The studies considered eligible were analyzed by an independent reviewer, who read the texts in their entirety. An Excel spreadsheet, prepared by the authors, was used to extract data from the literature included in the scoping review, containing specific information in accordance with the recommendations of the JBI Manual for Evidence Synthesis.

The data extracted from the literature were organized into two main categories: bibliographic information and study characteristics, including title, authorship, year of publication, country where the study was conducted, objective, study design, population and sample, results, and conclusions.

### Data analysis and presentation

The data were presented in charts and figures accompanied by a descriptive analysis summarizing the information extracted. These data were organized and categorized, considering the objective of the study review and the safe practices for administering intravenous drug therapy in hospitalized adult patients identified in the literature.

## RESULTS

Initially, 24,320 studies were identified. After applying the 10-year filter, 6,896 studies were found in the databases. After identifying and excluding duplicate studies, a total of 5,932 studies were obtained. These studies were evaluated by reading the titles and abstracts, excluding those that did not fit the theme.

To consider the grey literature, a search was conducted on the websites of health authorities such as WHO, CDC, MS, Anvisa, Infusion Nurses Society (INS), Institute for Safe Medication Practices (ISMP), and the Regional Nursing Council of São Paulo (COREN-SP) to gather scientific evidence on the topic, resulting in a sample of eight documents that were read in full and included in the study.

Thus, 33 records were read, including 25 scientific articles indexed in the selected databases and 8 from grey literature. Subsequently, 15 studies were excluded, 3 because they were not available in full and 12 because they did not meet the study’s eligibility criteria, with 18 studies included in the scoping review (Figure 1).

**Figure 1:**
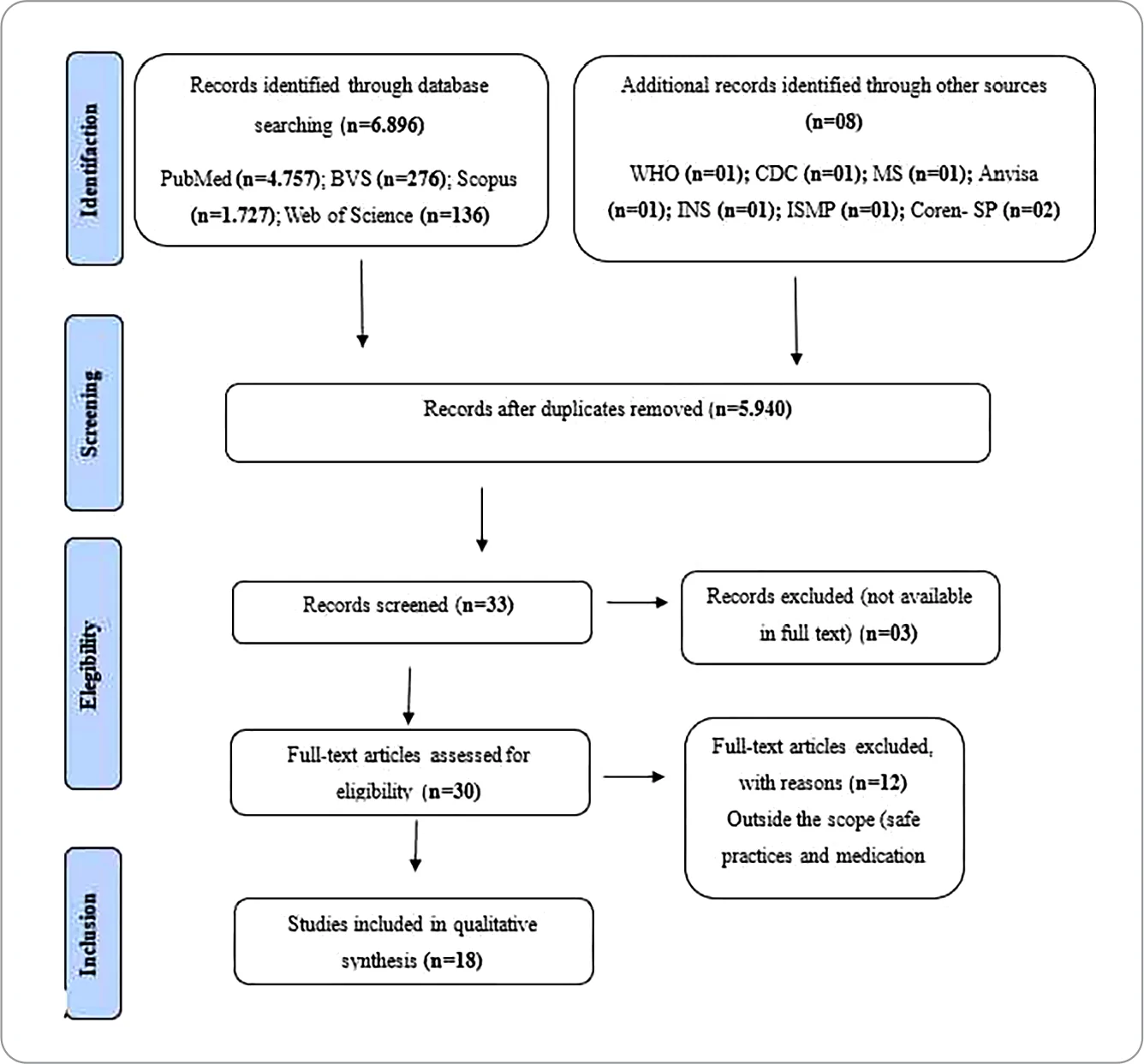
Flowchart for identifying and selecting primary studies included in the scoping review according to PRISMA-ScR, Rio das Ostras, Rio de Janeiro, Brazil, 2025

Based on the selection from databases and gray literature, the sample for this review consisted of 18 studies published between 2015 and 2024, four of which were from 2020. Regarding the countries of publication, there is a predominance of studies in the United States of America (n=4) and Brazil (n=4), followed by England (n=2) and Italy (n=2). Regarding study design, there was a predominance of cross-sectional studies (n=4), observational studies (n=3), and technical documents (n=3) (Chart 2).

**Chart 2:** Characterization of studies according to title, authors, year of publication, country where the study was conducted, objective, and study design, Rio das Ostras, Rio de Janeiro, Brazil, 2025

	Title	Authors	Year	Country	Objective	Study design
E1	Erros and discrepancies in the administration of intravenous infusions: a mixed methods multihospital observational study	Lyons *et al.* ^(21)^	2018	England	To determine the prevalence, types, and severity of errors and discrepancies in infusion administration in English hospitals and explore sources of variation, including the contribution of smart pumps.	Observational Study
E2	Standardization and Chemical Characterization of Intravenous Therapy in Adult Patients: A Step Further in Medication Safety	Manrique-Rodríguez *et al.* ^(22)^	2020	Spain	Standardize the dilutions of the most commonly used non-oncology drugs in hospitalized adult patients and characterize these dilutions in terms of their pH, osmolarity, and cytotoxic nature to complement current knowledge in order to guide the selection of the most appropriate vascular access for each patient.	Observational and Prospective Study
E3	Nurses’ atitudes and knowledge of peripherally inserted central catheter maintenance in primary hospitals in china: a crosssectional survey	Xu *et al.* ^(23)^	2020	China	To assess nurses’ attitudes and knowledge about PICC maintenance in primary care hospitals.	Cross-sectional study
E4	Frontline nursing staff’s perceptions of intravenous medication administration: the first step toward safer infusion processes - a qualitative study	Uramatsu *et al.* ^(24)^	2024	Japan	Identify the mental models used by frontline nurses during the intravenous drug administration process, which may influence their interactions with patients and medication processes.	Prospective qualitative study
E5	Medication errors in the emergency department: knowledge, attitude, behavior, and training needs of nurses	Simone *et al*.^(25)^	2018	Italy	Describe the knowledge, attitudes, behaviors, and training needs regarding intravenous drug therapy management of the nursing staff in the emergency room of the Umberto I polyclinic in Rome, to prevent EMS.	Descriptive cross-sectional study
E6	Examination of Nursing drug administration practices via central venous catheter: An observational study	Arslan *et al*.^(26)^	2019	Turkey	Determine the level of compliance with the steps of the intravenous medication administration procedure associated with central venous catheter (CVC) access for bolus infusion in intensive care units.	Observational study
E7	*Práctica de enfermería en la dilución y administración de antibióticos intravenosos en un hospital público del centro de México*	Trujillo *et al.* ^(27)^	2024	Mexico	Describe nursing practice in the dilution and administration of intravenous antibiotics in adult patients in the Medical Clinic Department of a public hospital in central Mexico.	Quantitative, descriptive, and cross-sectional study
E8	Knowledge, behaviours, training and attitudes of nurses during preparation and administration of intravenous medications in intensive care units (ICU). A multicenter Italian Study	Muzio *et al.* ^(28)^	2017	Italy	To describe the knowledge, attitudes, behaviors, and training needs of Italian nurses working in ICUs regarding the use of intravenous drugs and to identify strategies that nurses can adopt to prevent medication errors.	Cross-sectional study
E9	Untangling Infusion Confusion: A Comparative Evaluation of Interventions in a Simulated Intensive Care Setting	Pinkney *et al.* ^(29)^	2019	Canada	Assess the impact of interventions on preventing errors in the identification and disconnection of intravenous infusions.	Experimental study
E10	Nurses as a source of system-level resilience: secondary analysis of qualitative data from a study of intravenous infusion safety in English hospitals	Vos *et al.* ^(30)^	2020	England	Conduct a more detailed analysis of ECLIPSE data to explore the ways in which nurses contribute to system-level resilience in relation to intravenous infusion safety.	Mixed study
E11	Safe Practice Guidelines for Adult IV Push Medications	ISMP^(31)^	2015	USA	Identify risks, examine current evidence, and make recommendations for safer practices associated with the preparation and administration of intravenous injections in adults.	Guideline
E12	Prevention For Outpatient Settings: Minimum Expectations for Safe Care	CDC^(32)^	2016	USA	1) Provide basic infection prevention recommendations for outpatient settings; 2) Reaffirm standard precautions as the basis for preventing the transmission of infectious agents during patient care in all healthcare settings; 3) Provide links to complete guidelines and reference documents, which readers can consult for more detailed information and recommendations.	Technical document
E13	*Medidas de Prevenção de Infecção Relacionadas à Assistência à Saúde*	Anvisa^(33)^	2017	Brazil	Contribute to reducing the incidence of HAIs in healthcare services by providing the main practical preventive measures appropriate to the Brazilian reality.	Technical document
E14	Medication safety for look-alike, sound-alike medicines	WHO^(34)^	2023	Switzerland	Address important aspects related to medication safety.	Technical document
E15	Infusion Therapy Standards Of Pratice	INS- Nickel et al.^(35)^	2024	USA	-	Book
E16	*Anotações de Enfermagem*	COREN- SP^(36)^	2022	Brazil	-	Book
E17	*PARECER COREN-SP Nº 007/2023*	COREN-SP^(37)^	2023	Brazil	Performance of the nursing team in intravenous therapy.	Technical Opinion
E18	*Protocolo de segurança na prescrição, uso e administração de medicamentos*	MS^(38)^	2020	Brazil	Promote safe practices in the use of medicines in healthcare facilities.	Protocol

The scientific evidence found in the analyzed literature on safe practices for intravenous drug administration was mapped and grouped into five stages: 1) Aseptic techniques; 2) Preparation; 3) Labeling; 4) Administration; 5) Recording (Chart 3).

**Chart 3:** Results according to the categories analyzed in the scientific literature, Rio das Ostras, Rio de Janeiro, Brazil, 2025

Category	Empirical Studies	Technical documents
Aseptic Techniques	E3; E5; E6; E8	E11; E12; E13; E15; E17; E18
Preparation	E1; E2; E4; E5; E6; E8; E10	E11; E12; E13; E15; E18
Labeling	E1; E9	E11; E14; E15
Administration	E1; E3; E4; E5; E6; E7; E8; E10	E11; E14; E15; E17; E18
Recording		E15; E16; E18

The literature reviewed addresses safe practices in intravenous drug therapy. National and international evidence often points out that aseptic techniques are extremely important and permeate the various stages of the procedure. Hand hygiene, disinfection of vial diaphragms and medication ampoule necks, as well as disinfection of vascular access connectors, are highlighted.

Hand hygiene before and after the procedure was considered a fundamental measure and one of the most frequent pieces of evidence of safe practice in the preparation and administration of intravenous drug therapy that promotes patient safety^(25,26,28,31,32,37,38)^.

The diaphragms of vials and the necks of medication ampoules must also be disinfected before the drugs are drawn up, during the medication preparation stage, thus ensuring that aseptic technique is maintained throughout the process. Similarly, it is necessary to clean the venous access connectors during the administration stage, but before it is performed, in order to avoid contamination^(23,26,31-33,35,37)^.

The measures most frequently discussed in the literature on medication preparation were double-checking the prescription and verifying all important information such as the name of the medication, dose, concentration, volume, expiration date, route of administration, frequency, and rate of administration^(21,24-26,31,35)^. In the third stage, labeling, the correct identification of medications and devices associated with their administration is an essential component of patient safety in medication practice, promoting patient-centered care. Thus, labeling must be performed immediately, without interruption, in order to avoid administration errors or improper exchanges^(21,29,34,35)^.

With regard to the administration of intravenous drug therapy, the literature highlights the importance of checking prescriptions, verifying allergies, informing patients about their medication, and confirming their identity. In addition, the analyzed literature repeatedly emphasizes the need to assess vascular access and verify its permeability^(23,26,31,35,37)^. Washing the infusion line between different medications and after administration was considered a technique frequently used as a safety measure^(25-27,35,36)^. It also highlighted the recording of administration, patient appeals, and adverse events, such as signs and symptoms observed, and actions taken^(35-38)^. (Figure 2).

**Figure 2:**
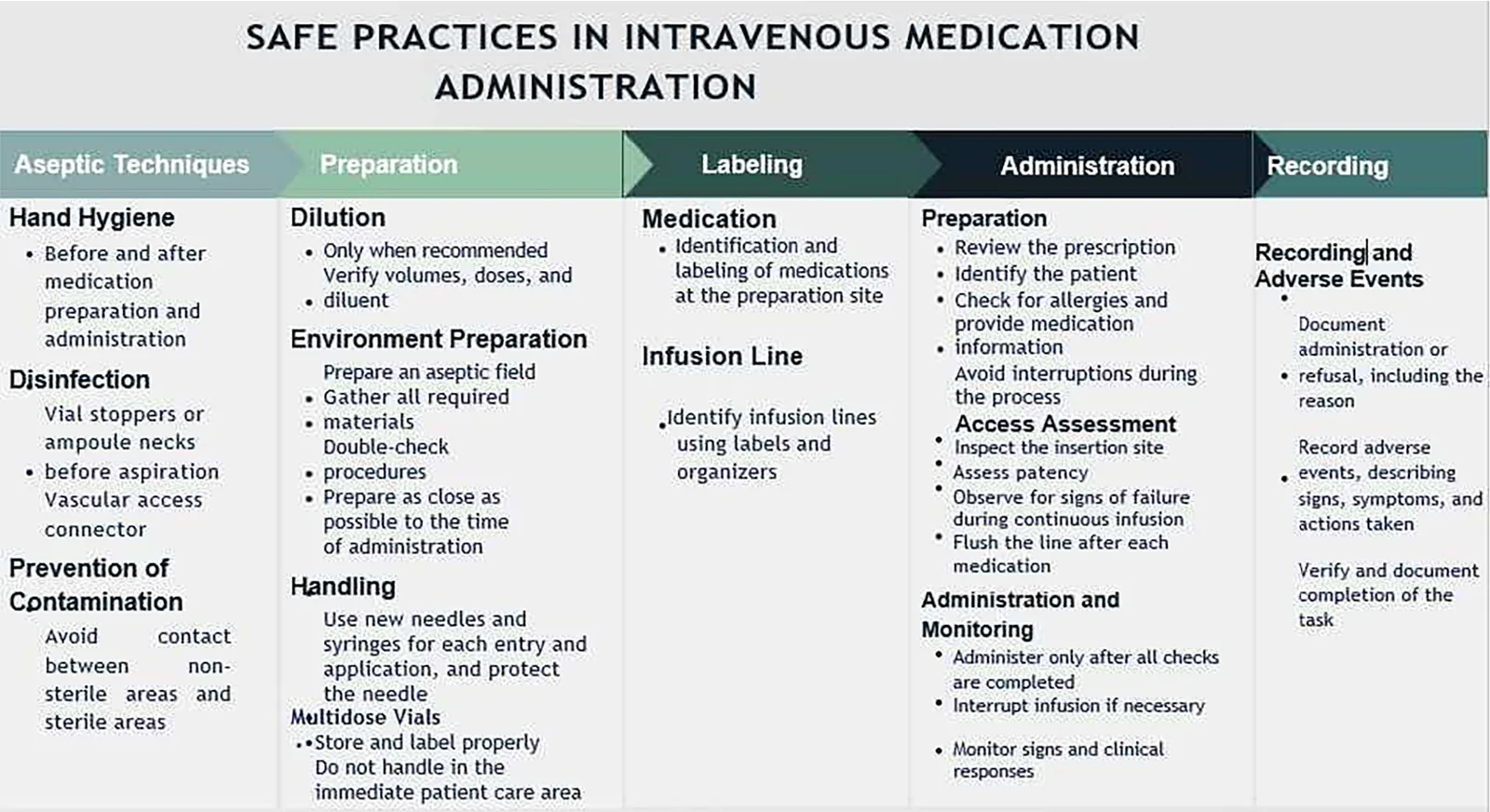
Safe practices in intravenous drug therapy, Rio das Ostras, Rio de Janeiro, Brazil, 2025

## DISCUSSION

The scoping review identified safe practices in intravenous drug therapy in hospitalized adult patients. These practices include technical rigor and compliance with aseptic protocols at all stages of the process, from preparation to recording, thus contributing directly to risk reduction and helping to prevent healthcare-associated infections, promoting patient safety.

The adoption of aseptic techniques in the pre-administration, administration, and post-administration of intravenous drug therapy is essential for the prevention of healthcare-associated infections. In this scoping review, the following were highlighted hand hygiene before, during, and after the administration of intravenous drug therapy - cross-sectional study (E5); CDC technical document (E12); WHO technical document (E14). These findings from the scoping review corroborate the results of a study conducted in a large hospital in a city in the interior of the state of São Paulo in medical and surgical clinics, in which of the 385 observations made of the intravenous drug administration process, hand hygiene was observed before and after the preparation of the medication in all cases^(12)^, standard that must be routinely followed in healthcare institutions.

Hand hygiene is considered a safe practice in intravenous drug therapy in hospitalized adult patients, and poor adherence to it is one of the main causes associated with healthcare-associated infections (HAIs)^(39)^. Despite recommendations from official bodies such as the Ministry of Health and ANVISA (technical documents E13 and E18), hand hygiene remains a challenge in nursing practice in Brazil and internationally, even though it is a simple, low-cost technique that is effective in preventing and controlling HAIs. A study conducted in a private hospital in Bahia identified low adherence to hand hygiene as one of the errors in the preparation and administration of intravenous medications. Similarly, an international study conducted in Ethiopia observed that only 54 (24.0%) of professionals performed hand hygiene when necessary^(7,39,40)^.

Aseptic measures, such as disinfecting the diaphragm of vials and the necks of medication ampoules during preparation and cleaning venous access connectors before administration - cross-sectional study (E3); technical document (E11), are safe practices evidenced in the scoping of the analyzed literature and that have also been neglected, yet are essential to ensure the maintenance of aseptic technique during IV drug therapy in adults. These measures, although simple, prevent the spread of HAIs, given that neglecting this step can lead to contamination of the infusion site and the patient’s skin, thus increasing the risk of phlebitis and bloodstream infection. These findings are consistent with a study conducted at a referral hospital for infectious diseases in Fortaleza, which highlighted that among 111 observations of drug preparation and administration, only 13 (11.7%) performed disinfection of the ampoule or vial^(40,41)^.

During the preparation stage, the need for double-checking the medical prescription and verifying all important information was highlighted - prospective study (E4); observational and prospective study (E2), as safe practices in intravenous drug therapy in hospitalized adult patients. When these practices are neglected by professionals, they can lead to medication errors, as identified in a study conducted in a hospital in the state of Bahia, Brazil, where the error rate in the preparation of intravenous medications was 60% and one of the main errors found during the preparation of medications was the incorrect identification of the prepared medication. thus highlighting the importance of double-checking to verify all necessary information and ensure patient safety^(40)^. It is worth noting that hospitals play a fundamental role in promoting patient safety by incorporating documents or protocols into the institution that present information on dilution, reconstitution, and concentration of medications, as well as training and capacity building for healthcare professionals^(42)^, thus reinforcing the shared responsibility between institutions and professionals in promoting patient safety.

In the labeling stage, the correct and immediate identification of medications and devices associated with their administration-technical documents (E11; E15); observational study (E1); experimental study (E9)-are essential components for patient safety in intravenous drug therapy in hospitalized adult patients, promoting patient-centered practice. It should be noted that the label must contain complete information such as medication, unit, dose, type of diluent, total volume to be administered, time, and name of the person who prepared it^(42-44)^. Identifying medications through labeling after preparation is an important safety barrier before administering the medication. It is a low-cost strategy that organizes the work process, preventing patients from being affected by possible errors, such as medication mix-ups and drug allergy warnings, which can occur at all stages of intravenous drug therapy^(42-44)^.

The administration stage involves several essential steps aimed at preventing adverse events and ensuring safe practices in intravenous drug therapy in hospitalized adult patients, ranging from reviewing the medical prescription, identifying the patient, checking for possible allergies, providing information about what is being administered, to clarifying any questions - prospective qualitative study (E4); observational study (E6); cross-sectional study (E8). These safe practices compiled in this scoping review, although simple and based especially on reviews, verifications, and checks, are often neglected or not performed, as can be seen in a study conducted at a referral hospital in the regional state of Amhara, Ethiopia, which highlighted that of the 109 doses of medication administered, only 11% involved safe practices. The most significant errors observed at this stage were: not clarifying and/or informing the patient about what is being administered and not assessing the patient after drug administration, which is an essential moment for identifying possible adverse events^(45)^.

The identification of patients with at least two indicators, such as full name and medical record number, was also pointed out in another study as one of the main barriers, thus demonstrating, as in the present review, the need for correct patient identification at the time of drug administration, ensuring safe practice in intravenous drug therapy in hospitalized adult patients. A study conducted in a hospital in the interior of São Paulo found that 79% of the patients interviewed reported that there was no type of verification of their name before the administration of the medication. This evidence demonstrates the importance of adhering to this good practice for safety in care, as it contributes to reducing the rate of preventable harm^(42,46)^. Thus, it is noted that the adoption of safe practices in intravenous drug therapy in hospitalized adult patients, related to double-checking the prescription at the time of administration, verifying patient identification, and the need to be aware of patient allergies, contributes to the reduction of medication errors^(47)^.

The literature review showed that safe practices in intravenous drug therapy in hospitalized adult patients include assessing the vascular access site and checking its patency before administering intravenous drug therapy, as well as flushing the infusion line after each medication administration - cross-sectional study (E7); mixed study (E10); technical opinion (E17). Thus, performing the flushing technique, washing the infusion line before, between, and after medications, is an essential care measure for the nursing team, which aims to assess and maintain the patency of vascular access, preventing its obstruction and contributing to patient safety. The literature reviewed highlights that flushing should be performed with 0.9% saline solution and, despite its recommendation in guidelines, the practice is still permeated by low adherence, as identified in a study conducted in a hospital in the interior of São Paulo, Brazil, in which only 4.81% of administrations had this technique performed^(12,48)^. Such discrepancies between what is recommended and what is actually practiced raise discussions about the barriers that hinder the incorporation of safe practices into daily care and the need for educational and institutional strategies for their consolidation.

The recording stage is a safe practice that must be performed in intravenous drug therapy in hospitalized adult patients, according to the scoping of the literature, since recording medication refusals, complications, adverse events, and checking the medication administered contributes to the traceability of care, therapeutic continuity, and the prevention of errors related to medication administration - book (E16); protocol (E18). Corroborating the safe practices found in this scoping review, a study conducted in a hospital in the city of Fortaleza, Brazil, found that 92 (82.8%) of nursing professionals record and check after administration, however, only 20 (18%) recorded the complaints and adverse reactions presented by the patient^(41,47)^, demonstrating that despite performing a safe practice, professionals still do not perform it completely.

The safe practices highlighted in this scoping review are part of a set of measures that are necessary in all IV drug therapy administration; however, the particularities of each stage of the patients’ lives must be considered. A study conducted on this topic in pediatric patients presents important particularities to be considered for this specific audience, which was not the population chosen for this review, such as physiological immaturity, the unavailability of drug formulations suitable for this age group, and the subdoses that need to be administered, which are closely related to the patient’s weight and age^(49)^.

In the national context, nursing professionals face several barriers to safe intravenous drug therapy practices in hospitalized adult patients, such as work overload, communication failures between teams, scarcity of technological resources, and lack of an institutional culture focused on safety, factors that contribute to the occurrence of inappropriate and potentially unsafe practices^(50)^. Internationally, these difficulties are also widely recognized and have been addressed by the WHO, which includes the safe administration of medications among its global patient safety goals^(50)^. Several countries have been implementing such guidelines and investing in support technologies, such as computerized prescription and electronic patient identification systems, in addition to promoting an organizational culture based on incident reporting and continuing education^(50)^. Thus, although institutional and sociocultural contexts vary, there is a universal need to strengthen the implementation of safe practices in the administration of intravenous drug therapy^(50)^. Therefore, mapping the scientific evidence provides a current overview that can contribute to reducing a gap in nursing practice and safe care in intravenous drug therapy in adults.

### Study Limitations

This scoping review has limitations, such as the absence of specific descriptors for the topic, which may have led to a limited sample and the exclusion of relevant studies. In addition, it should be noted that some articles were not included because they were not available in full.

The methodological quality of the included studies was not assessed; thus, the practices identified as safe were described based on the recommendations of the studies themselves, without analyzing the level of evidence. The heterogeneity of the studies included in the review, in terms of institutional contexts, professionals involved, types of medications, and technologies used, limits the generalization of the findings.

### Contributions to the Field

This study offers significant contributions to nursing practice by highlighting the importance of adopting safe practices in intravenous drug therapy in the hospital setting. By mapping this practice, which promotes patient safety, the study reinforces the strategic role of the nursing team in preventing medication errors. The evidence found contributes not only to strengthening the quality of care provided, but also serves as a valuable resource for nursing education, management, and professional practice.

## CONCLUSIONS

This study contributes to mapping scientific evidence on safe practices in intravenous drug therapy in hospitalized adult patients, providing relevant insights for improving patient safety. The main measures identified include the use of aseptic techniques, double-checking prescriptions, standardized labeling of medications, and systematic recording of administration and possible adverse reactions. These actions represent critical steps in the medication process and are decisive for the quality and safety of care.

The findings reinforce the need for continuous training of nursing teams, adoption of institutional protocols, and strengthening of the culture of safety in the hospital setting. The importance of implementing institutional and regulatory policies that standardize safe practices, promote systematic audits, and ensure adequate working conditions for the safe administration of medications was evident. Thus, this study supports both clinical practice and the formulation of policies aimed at patient safety and quality of care.

## Data Availability

The research data are available in a repository: https://osf.io/hv7g2.
