# Supplementary material for: Safe Practices in Intravenous Drug Therapy in Hospitalized Adult Patients: Scoping Review
Source: Rev Bras Enferm. 2026 Sep 28;79:e20250384. doi: 10.1590/0034-7167-2025-0384 (PMC13623393; doi:10.1590/0034-7167-2025-0384)
Supplement: Translation

# Translation: Práticas Seguras na Terapia Medicamentosa Intravenosa em Pacientes Adultos Hospitalizados: Revisão de Escopo

**DOI:** 10.1590/0034-7167-2025-0384pt

**Authors:** Hevelyn dos Santos da Rocha, Milena Cristina Couto Guedes, Maithê de Carvalho e Lemos Goulart, Fernanda Garcia Bezerra Góes, Asaphe Stenio Lima dos Santos, Fernanda Maria Vieira Pereira-Ávila

**Affiliations:** I Universidade Federal Fluminense. Rio das Ostras, Rio de Janeiro, Brasil

**Keywords:** Infusões Intravenosas, Cuidados de Enfermagem, Enfermagem, Hospitais, Adulto.

## Abstract

**Objetivos:**

mapear as práticas seguras na terapia medicamentosa intravenosa em pacientes adultos hospitalizados descritas na literatura científica.

**Métodos:**

revisão de escopo conforme JBI, com a pergunta: “Quais as práticas para administração segura de terapia medicamentosa intravenosa em adultos hospitalizados?”. Realizou-se as buscas em quatro recursos informacionais e literatura cinzenta. Duplicatas foram removidas no *Endnote* e os estudos selecionados no *Rayyan*, conforme critérios de elegibilidade. Os dados foram apresentados segundo o *Preferred Reporting Items for Systematic Reviews and Meta-analyses Extension for Scoping Review*.

**Resultados:**

analisaram-se 18 estudos cujas evidências foram agrupadas nas etapas: 1) Técnicas assépticas; 2) Preparo; 3) Rotulagem; 4) Administração; 5) Registro. Destacou-se como práticas seguras: higiene das mãos, dupla checagem, identificação dos medicamentos e paciente, verificação da permeabilidade do acesso, registro da administração e eventos adversos.

**Conclusões:**

adoção de práticas seguras nas etapas da terapia medicamentosa intravenosa constitui estratégias fundamentais para segurança do paciente adulto hospitalizado.

## INTRODUÇÃO

A segurança do paciente é definida pela Organização Mundial da Saúde (OMS) como a ausência de danos evitáveis e redução de danos desnecessários associados aos cuidados de saúde^(1)^. Nesse contexto, o erro na terapia medicamentosa afeta diretamente a segurança do paciente, sendo caracterizado como qualquer evento prevenível que pode ocasionar ou resultar no uso inadequado de medicamentos, bem como em danos ao paciente, durante o período em que a terapêutica estiver sob a responsabilidade do profissional de saúde, paciente ou consumidor. Tais erros podem estar vinculados à prática profissional, ao produto, procedimento e sistemas de saúde, abrangendo desde a prescrição até o monitoramento e uso dos medicamentos^(2)^.

De acordo com OMS, as práticas de medicação inseguras e erros de medicação são um dos principais fatores de danos ao paciente, afetando a segurança de 1 a cada 30 pacientes, sendo a maioria desses danos evitáveis. Além disso, estima-se que mundialmente os custos associados a esses erros são de 42 milhões de dólares anuais. Com isso, em 2017, a OMS definiu a medicação sem dano como terceiro desafio global para segurança do paciente^(1,3)^. No contexto internacional, os erros de medicação causam anualmente 1,3 milhões de lesões^(4)^, 9% dos erros de medicação em hospital ocorrem na etapa da administração de medicamento^(5)^ e cerca de 25% dos eventos encontrados nos prontuários estão relacionados à terapia medicamentosa^(6)^.

O cenário brasileiro não é diferente, cerca de 30% das doses administradas em hospitais de diferentes regiões do país continham algum tipo de falha, sendo 1.500 erros relacionados à administração de medicamento em unidades de internação clínica^(2)^. Os erros ocorrem, predominantemente, na fase de preparo e administração da medicação e podem estar associados a diversos fatores como unidades hospitalares sobrecarregadas, fadiga do profissional, condições ambientais inadequadas e conhecimento insuficiente^(5,7-9)^.

As consequências dos erros na terapia medicamentosa podem ser exacerbadas a depender de diversos fatores, entre eles, a idade do paciente. Destaca-se que adultos entre 18 e 59 anos estão no processo de envelhecimento e, portanto, mais suscetíveis às interações medicamentosas e danos. Uma revisão integrativa da literatura, que analisou 22 artigos, entre 2018 e 2023, sobre segurança do paciente e erros na terapia medicamentosa com o mesmo grupo da população, identificou que os principais erros também ocorrem no preparo e administração, com destaque para omissão ou erro de doses, falhas na preparação e rotulagem inadequada^(10,11)^.

Tais aspectos podem ser ainda mais graves quando administradas medicações parenterais, cuja ação é sistêmica, como a terapia medicamentosa intravenosa que consiste na infusão de líquidos e medicamentos pela via intravenosa (IV). Nesse caso, os eventos adversos oriundos dos erros frequentemente estão associados à clínica e à infecção da corrente sanguínea (ICS) pela infusão de soluções contaminadas^(5,12)^, no acesso vascular do paciente, que pode ser periférico ou central. Estima-se que 80% dos pacientes hospitalizados utilizam esses dispositivos, o que aumenta o risco de eventos adversos^(13,14)^.

Na terapia medicamentosa IV, embora a equipe multidisciplinar esteja envolvida no processo de prevenção dos erros de medicação e promoção da segurança do paciente, a equipe de enfermagem possui um papel de extrema importância. Considerando que os profissionais de enfermagem estão presentes em todo o processo e são a última barreira para evitar possíveis danos ao paciente, é fundamental garantir o preparo, administração e monitoramento da terapia medicamentosa com segurança^(7,15,16)^.

Ainda, é necessário implementar estratégias e processos, além de fortalecer a cultura de segurança do paciente^(16)^, para isso é fundamental que a enfermagem domine farmacocinética, farmacovigilância e todas as etapas da terapia medicamentosa IV, considerando suas especificidades e riscos ao paciente adulto^(17,18)^. A terapia medicamentosa intravenosa constitui uma prática amplamente empregada no ambiente hospitalar, especialmente no cuidado a pacientes adultos, sendo essencial para o tratamento de diversas condições clínicas. Contudo, apesar de sua reconhecida importância na prática assistencial, persistem lacunas referentes à ausência de padronização nos processos envolvidos, o que compromete a uniformização das práticas desde o preparo e a administração dos fármacos até o monitoramento dos efeitos terapêuticos e a identificação de possíveis reações adversas.

O mapeamento das práticas seguras na terapia medicamentosa IV, portanto, faz-se imprescindível, de modo colaborar com a prática baseada em evidência e contribuir para a tomada de decisão clínica informada, baseada nas evidências atuais e diretrizes nacionais e internacionais sobre o tema. Além disso, as evidências disponíveis na literatura apresentam-se dispersas e abordam as práticas de forma fragmentada, frequentemente restritas a etapas isoladas do processo terapêutico ou a contextos específicos de atuação. Nesse sentido, uma revisão de escopo mostra-se pertinente por possibilitar a síntese e a integração dessas informações, reunindo, em um único estudo, o conjunto de práticas seguras relacionadas à terapia medicamentosa intravenosa em pacientes adultos hospitalizados.

Dessa forma, a presente revisão de escopo pode contribuir para qualificar a prática profissional, reforçando o papel da equipe de enfermagem na prevenção dos erros de medicação e na promoção da segurança do paciente, além de contribuir para a construção e/ou atualizações de protocolos clínicos, orientar programas de capacitações profissionais e fomentar novas pesquisas na área, contribuindo para o fortalecimento da cultura de segurança do paciente. Foi realizada uma busca preliminar em 25 de abril de 2025 no *Prospero Systematic review* e *Open Science Framewor* (OSF), contudo não foram identificadas outras revisões ou protocolos de revisões atuais ou em andamento sobre a temática. Destaca-se que existem estudos primários sobre o tema garantindo a exequilidade desta revisão de escopo.

## OBJETIVOS

Mapear as práticas seguras na terapia medicamentosa intravenosa em pacientes adultos hospitalizados descritas na literatura científica.

## MÉTODOS

### Protocolo e Registro

Trata-se de uma revisão de escopo elaborada de acordo as diretrizes do JBI^(19)^, com objetivo de assegurar a transparência e a qualidade, conforme as recomendações contidas no *Preferred Reporting Items for Systematic Reviews and Meta-Analyses extension for Scoping Reviews* (PRISMA-ScR)^(20)^. Seguiu-se as etapas propostas pelo método, que consistiram em: definir e alinhar o objetivo de pesquisa; estabelecer critérios de inclusão com objetivo e a questão da pesquisa; descrever a abordagem planejada para a busca, seleção e extração de dados; realizar a busca, seleção, extração e análise das evidências; apresentar os resultados obtidos; sintetizar as evidências em relação ao objetivo da revisão de escopo, elaborando conclusões e destacando as implicações decorrentes da descoberta. A revisão de escopo foi devidamente registrada na plataforma OSF, DOI: 10.17605/OSF.IO/HV7G2.

### Pergunta de revisão

Para a construção da pergunta de pesquisa, utilizou-se a estratégia metodológica baseada no acrônimo PCC - P (População), C (Conceito) e C (Contexto). Na qual P (população) consistiu em: pessoas adultas; C (conceito), isto é: práticas seguras relacionadas à terapia medicamentosa intravenosa; C (contexto), ou seja: pacientes adultos que estejam internados no ambiente hospitalar. A partir desses elementos, formulou-se a seguinte pergunta de pesquisa: “Quais são as práticas para administração segura de terapia medicamentosa intravenosa em pacientes adultos hospitalizados?”.

### Critérios de elegibilidade

Para a contemplação da população, conceito e contexto, foram incluídos estudos que abordem práticas seguras e erros relacionados à administração de terapia medicamentosa intravenosa em indivíduos adultos no âmbito hospitalar. Foram considerados estudos que abordem os erros, tendo em vista que o acontecimento desses eventos contribui para direcionar ações de práticas seguras no ambiente de saúde.

O estudo adotou como critério de inclusão o recorte temporal dos últimos 10 anos (2015-2025). Essa restrição temporal foi estabelecida com objetivo de garantir a inclusão de estudos recentes que refletem as práticas atuais, diretrizes e avanços científicos na temática. Não foi considerado local ou idioma dos estudos.

Os artigos duplicados foram excluídos e contabilizados apenas uma vez. Além disso, foram excluídos estudos que apresentaram diretrizes específicas para manejo seguro e eficaz de medicações que são utilizadas com maior frequência em setores específicos do ambiente hospitalar, como por exemplo drogas vasoativas, utilizadas na unidade de terapia intensiva.

### Fontes de informação

Foram considerados desenhos de estudos experimentais e quase-experimentais, ensaios clínicos (randomizados ou não), séries temporais, estudos epidemiológicos, observacionais, transversais, analíticos, de coorte prospectivos ou retrospectivos, séries de casos, relatos de casos individuais e literaturas cinzentas relevantes, como diretrizes clínicas, manuais e documentos de organizações. Abarcou-se, ainda, pesquisas qualitativas e revisões sistemáticas. Foram excluídos estudos tipo carta, comentários, editoriais, artigos de opinião de especialistas e resumos de eventos. Espera-se o retorno de materiais e evidências diversas para a construção do escopo relativo ao tema.

### Estratégia de Busca

As buscas foram realizadas nos recursos informacionais: *Medical Literature Analysis and Retrieval System Online* (Medline) via PubMed, Biblioteca Virtual em Saúde, *Web of Science* e *Scopus,* acessadas por meio do Portal da Coordenação de Aperfeiçoamento de Pessoal de Nível Superior (CAPES). Além disso, para a contemplação da literatura cinzenta, foram considerados documentos de autoridades de saúde com Organização Mundial de Saúde (OMS), *Centers for Disease Control and Prevention* (CDC), Ministério da Saúde (MS), Agência Nacional de Vigilância Sanitária (ANVISA) disponíveis em suas bibliotecas virtuais.

A estratégia de busca inicial foi elaborada com auxílio de uma bibliotecária, a busca e a seleção dos estudos foram realizadas em três etapas distintas: 1- Identificação de palavras-chave em títulos e resumos; 2- Utilização de palavras-chave para busca em uma base de dados, sendo realizada na base de dados a PubMed; 3- Realização da busca nas bases de dados estabelecidas e aplicação dos critérios de elegibilidade. As etapas um e dois contribuíram na construção da estratégia/expressão de busca a ser utilizada na terceira etapa. Inicialmente, foram analisadas palavras presentes nos títulos e resumos, bem como termos de assuntos que descrevem os artigos. Posteriormente, as palavras-chave e os termos de assuntos identificados foram empregados na pesquisa completa na base de dados. Por fim, a busca foi conduzida com descritores dos vocabulários controlados no idioma inglês, do *Medical Subject Headings Section* (MeSH) com operador booleano AND e OR, associando as palavras-chave e suas variações nos campos de títulos, resumos e assunto do documento. As buscas foram realizadas em 08 de maio de 2025 (Quadro 1).

**Quadro 1:** Registro das estratégias de buscas para recuperação das publicações nas bases de dados, Rio das Ostras, Rio de Janeiro, Brasil, 2025

Registro de buscas			
**Fonte de Informação**	**Estratégia de Busca**	**Resultado** **Sem filtro**	**Resultado** **Com filtro 10 anos**
MEDLINE/ PubMed	*((Adult) AND (“Infusions, Intravenous”[mesh] OR “Intravenous Infusions” OR “Infusion, Intravenous” OR “Intravenous Infusion” OR “Intravenous Drip” OR “Drip, Intravenous” OR “Drip Infusions” OR “Drip Infusion” OR “Infusion, Drip” OR “Infusions, Drip”)) AND (Hospitals*)	18.603	4.757
BVS	*(adult) AND (“Infusions, Intravenous” OR “Intravenous Infusions” OR “Infusion, Intravenous” OR “Intravenous Infusion” OR “Intravenous Drip” OR “Drip, Intravenous” OR “Drip Infusions” OR “Drip Infusion” OR “Infusion, Drip” OR “Infusions, Drip”) AND (hospitals)*	832	276
Scopus	*TITLE-ABS-KEY (((adult) AND (“Infusions, Intravenous” OR “Intravenous Infusions” OR “Infusion, Intravenous” OR “Intravenous Infusion” OR “Intravenous Drip” OR “Drip, Intravenous” OR “Drip Infusions” OR “Drip Infusion” OR “Infusion,Drip” OR “Infusions, Drip”) AND (hospitals)))*	4.592	1.727
Web of Science	*((ALL=(Adult)) AND ALL=(“Infusions, Intravenous” OR “Intravenous Infusions” OR “Infusion, Intravenous” OR “Intravenous Infusion” OR “Intravenous Drip” OR “Drip, Intravenous” OR “Drip Infusions” OR “Drip Infusion” OR “Infusion, Drip” OR “Infusions, Drip”)) AND ALL=(Hospitals)*	293	136

### Seleção de fontes de evidências

Os resultados das buscas foram inicialmente exportados para *software* de gerenciamento de referência *Endnote Web*, em que foram removidas as duplicadas e, posteriormente, os resultados foram exportados para o *software Rayyan*, utilizado para organizar e conduzir o processo de seleção dos estudos que comporiam a revisão de escopo. Ambos os *softwares* com acesso online e gratuito.

A seleção das evidências foi conduzida por dois revisores, treinados, previamente, que analisaram os artigos individualmente, com cegamento de pares, em dispositivos distintos, sem interferência ou conhecimento sobre a seleção do outro revisor. Em caso de divergência entre os dois revisores no processo de seleção, as discordâncias foram resolvidas por meio de consenso entre os dois revisores.

Inicialmente, foi realizado a leitura dos títulos e resumos pelas duplas de revisores independentes, visando identificar estudos que atendessem os critérios de elegibilidade. Os estudos selecionados foram lidos na íntegra para a exclusão dos quais não respondem à pergunta de pesquisa desta revisão.

### Extração de dados

Os estudos considerados elegíveis foram analisados por um revisor independente, através da leitura integral dos textos. Para a extração dos dados da literatura incluída na revisão de escopo, utilizou-se uma planilha *Excel*, elaborada pelos autores, contendo informações específicas de acordo com as recomendações do JBI *Manual for Evidence Synthesis*.

Os dados extraídos da literatura foram organizados em duas categorias principais: informações bibliográficas e características dos estudos, que contemplam título, autoria, ano de publicação, país onde o estudo foi desenvolvido, objetivo, desenho do estudo, população e amostra, resultados e conclusões.

### Análise e apresentação dos dados

Os dados foram apresentados em quadros e figuras acompanhados de uma análise descritiva que sintetiza as informações extraídas. Os referidos dados foram organizados e categorizados, considerando o objetivo da revisão do estudo e as práticas seguras para administração da terapia medicamentosa intravenosa em pacientes adultos hospitalizados apontados na literatura.

## RESULTADOS

Identificou-se 24.320 estudos em um primeiro momento, após aplicação do filtro de 10 anos foram encontrados 6.896 estudos provenientes das bases de dados. Após a identificação e a exclusão dos estudos duplicados, obteve-se um total de 5.932 estudos. Estes estudos foram avaliados por meio da leitura dos títulos e resumos, excluindo-se aqueles que não estavam de acordo com a temática.

Para contemplação da literatura cinzenta, foi realizada pesquisa no site das autoridades de saúde como OMS, CDC, MS, Anvisa, *Infusion Nurses Society* (INS), *Institute for Safe Medication Practices* (ISMP) e Conselho Regional de Enfermagem de São Paulo (COREN-SP) para levantamento de evidências cientificas sobre a temática, assim obteve-se uma amostra de oito documentos que foram lidos na íntegra e incluídos no estudo.

Desse modo, procedeu-se à leitura de 33 registros, sendo 25 artigos científicos indexados nas bases de dados selecionadas e 8 de literatura cinzenta, na sequência foram excluídos 15 estudos, sendo 3 por não estarem disponíveis na íntegra e 12 por não se enquadrarem nos critérios de elegibilidade do estudo, sendo incluídos 18 estudos na revisão de escopo (Figura 1).

**Figura 1:** Fluxograma para identificação e seleção dos estudos primários incluídos na revisão de escopo de acordo com o PRISMA-ScR, Rio das Ostras, Rio de Janeiro, Brasil, 2025

A partir da seleção nas bases de dados e na literatura cinzenta, a amostra desta revisão foi composta por 18 estudos publicados entre 2015 e 2024, sendo quatro do ano de 2020. Em relação aos países de publicação, há um predomínio de estudos nos Estados Unidos da América (n=4) e no Brasil (n=4), seguido de Inglaterra (n=2) e Itália (n=2). Em relação ao desenho do estudo observa-se predomínio de estudos transversais (n=4), observacionais (n=3) e documentos técnicos (n=3) (Quadro 2).

**Quadro 2:** Caracterização dos estudos segundo título, autores, ano de publicação, país de realização do estudo, objetivo e desenho do estudo, Rio das Ostras, Rio de Janeiro, Brasil, 2025

	Título	Autores	Ano	País	Objetivo	Desenho do estudo
E1	*Erros and discrepancies in the administration of intravenous infusions: a mixed methods multihospital observational study*	Lyons *et al.* ^(21)^	2018	Inglaterra	Determinar a prevalência, os tipos e a gravidade de erros e discrepâncias na administração de infusão em hospitais ingleses e explorar fontes de variação, incluindo a contribuição de bombas inteligentes.	Estudo Observacional
E2	*Standardization and Chemical Characterization of Intravenous Therapy in Adult Patients: A Step Further in Medication Safety*	Manrique-Rodríguez *et al.* ^(22)^	2020	Espanha	Padronizar as diluições dos medicamentos não oncológicos mais utilizados em pacientes adultos hospitalizados e caracterizar essas diluições quanto ao seu pH, osmolaridade e natureza citotóxica para complementar o conhecimento atual a fim de orientar a seleção do acesso vascular mais adequado para cada um.	Estudo Observacional e prospectivo
E3	*Nurses’ atitudes and knowledge of peripherally inserted central catheter maintenance in primary hospitals in china: a crosssectional survey*	Xu *et al.* ^(23)^	2020	China	Avaliar as atitudes e o conhecimento dos enfermeiros sobre manutenção do PICC em hospitais primários.	Estudo transversal
E4	*Frontline nursing staff’s perceptions of intravenous medication administration: the first step toward safer infusion processes - a qualitative study*	Uramatsu *et al.* ^(24)^	2024	Japão	Identificar os modelos mentais utilizados por enfermeiros da linha de frente durante o processo de administração de medicamentos intravenosos, que podem influenciar suas interações com os pacientes e os processos de medicação.	Estudo qualitativo prospectivo
E5	*Medication errors in the emergency department: knowledge, attitude, behavior, and training needs of nurses*	Simone *et al*.^(25)^	2018	Itália	Descrever o conhecimento, as atitudes, os comportamentos e as necessidades de treinamento sobre o manejo de terapia farmacológica intravenosa da equipe de enfermagem do pronto-socorro do policínico Umberto I de Roma, a fim de prevenir Ems.	Estudo descritivo transversal
E6	*Examination of Nursing drug administration practices via central venous catheter: An observational study*	Arslan *et al*. ^(26)^	2019	Turquia	Determinar o nível de conformidade com as etapas do procedimento de administração intravenosa de medicamentos associados ao acesso Cateter venoso central (CVC) para infusão em bolus em unidades de terapia intensiva.	Estudo observacional
E7	*Práctica de enfermería en la dilución y administración de antibióticos intravenosos en un hospital público del centro de México*	Trujillo *et al.* ^(27)^	2024	México	Descrever a prática de enfermagem na diluição e administração de antibióticos intravenosos em pacientes adultos no Departamento de Clínica Médica de um hospital público no centro do México.	Estudo quantitativo, descritivo e transversal
E8	*Knowledge, behaviours, training and attitudes of nurses during preparation and administration of intravenous medications in intensive care units (ICU). A multicenter Italian Study*	Muzio *et al.* ^(28)^	2017	Itália	Descrever o conhecimento, as atitudes, os comportamentos e as necessidades de treinamento dos enfermeiros italianos que trabalham em UTIs em relação ao uso de medicamentos intravenosos e identificar as estratégias que os enfermeiros podem adotar para prevenir a ocorrência de erros de medicação.	Estudo transversal
E9	*Untangling Infusion Confusion: A Comparative Evaluation of Interventions in a Simulated Intensive Care Setting*	Pinkney *et al.* ^(29)^	2019	Canadá	Avaliar o impacto das intervenções na prevenção de erros na identificação e desconexão de infusões intravenosas.	Estudo experimental
E10	*Nurses as a source of system-level resilience: secondary analysis of qualitative data from a study of intravenous infusion safety in English hospitals*	Vos *et al.* ^(30)^	2020	Inglaterra	Conduzir uma análise mais detalhada dos dados do ECLIPSE para explorar as maneiras pelas quais os enfermeiros contribuem para a resiliência em nível de sistema em relação à segurança das infusões intravenosas.	Estudo misto
E11	*Safe Practice Guidelines for Adult IV Push Medications*	ISMP^(31)^	2015	EUA	Identificar os riscos, examinar as evidências atuais e fazer recomendações para práticas mais seguras associadas à preparação e administração de injeções intravenosas em adultos.	Diretriz
E12	*Prevention For Outpatient Settings: Minimum Expectations for Safe Care*	CDC^(32)^	2016	EUA	1)Fornece recomendações básicas de prevenção de infecções para ambientes ambulatoriais; 2) Reafirmar as precauções padrão como base para prevenção de transmissão de agentes infecciosos durante o atendimento ao paciente em todos os ambientes de saúde; 3) Fornecer links para diretriz completas e documentos de referência, que os leitores podem consultar para obter informações mais detalhadas e recomendações.	Documento técnico
E13	Medidas de Prevenção de Infecção Relacionadas à Assistência à Saúde	Anvisa^(33)^	2017	Brasil	Contribuir para reduzir a incidência das IRAS em serviços de saúde, a partir da disponibilização das principais medidas preventivas práticas adequadas à realidade brasileira	Documento técnico
E14	*Medication safety for look-alike, sound-alike medicines*	WHO^(34)^	2023	Suíça	Abordar aspectos importantes relacionados à segurança da medicação	Documento técnico
E15	*Infusion Therapy Standards Of Pratice*	INS- Nickel et al.^(35)^	2024	EUA	-	Livro
E16	Anotações de Enfermagem	COREN- SP^(36)^	2022	Brasil	-	Livro
E17	PARECER COREN-SP Nº 007/2023	COREN-SP^(37)^	2023	Brasil	Atuação da equipe de Enfermagem na Terapia intravenosa.	Parecer Técnico
E18	Protocolo de segurança na prescrição, uso e administração de medicamentos	MS^(38)^	2020	Brasil	Promover práticas seguras no uso de medicamentos em estabelecimentos de saúde.	Protocolo

As evidências cientificas encontradas na literatura analisada, sobre práticas seguras para administração de medicamento intravenoso, foram mapeadas e agrupadas em cinco etapas: 1) Técnica assépticas; 2) Preparo; 3) Rotulagem; 4) Administração; 5) Registro (Quadro 3).

**Quadro 3:** Resultados de acordo com as categorias analisadas na literatura científica, Rio das Ostras, Rio de Janeiro, Brasil, 2025

Categoria	Estudos Empíricos	Documentos técnicos
**Técnicas Assépticas**	E3; E5; E6; E8	E11; E12; E13; E15; E17; E18
**Preparo**	E1; E2; E4; E5; E6; E8; E10	E11; E12; E13; E15; E18
**Rotulagem**	E1; E9	E11; E14; E15
**Administração**	E1; E3; E4; E5; E6; E7; E8; E10	E11; E14; E15; E17; E18
**Registro**		E15; E16; E18

A literatura analisada aborda as práticas seguras na terapia medicamentosa intravenosa, evidências nacionais e internacionais frequentemente apontam que as técnicas assépticas são de extrema importância e permeiam as diversas etapas do procedimento. Destacam-se a higiene das mãos, desinfecção dos diafragmas dos frascos e gargalos das ampolas de medicação, como também a realização da desinfecção dos conectores dos acessos vasculares.

A higiene das mãos antes e após o procedimento foi considerada uma medida fundamental e umas das evidências mais frequentes de prática segura no preparo e administração de terapia medicamentosa intravenosa que promove a segurança do paciente^(25,26,28,31,32,37,38)^, tanto quanto a desinfecção do diafragma de frascos e gargalos de ampolas de medicações, que devem ser feitas antes da aspiração dos fármacos, ainda na etapa de preparo das medicações, garantindo a manutenção da técnica asséptica durante o processo. Da mesma forma, é necessário limpar os conectores do acesso venoso na etapa da administração, sobretudo antes da sua realização, a fim de evitar a contaminação^(23,26,31-33,35,37)^.

As medidas mais abordadas na literatura acerca do preparo da medicação foram: dupla checagem da prescrição, conferir todas as informações importantes como nome do medicamento, dose, concentração, volume, validade, via, frequência e taxa de administração^(21,24-26,31,35)^. Na terceira etapa, rotulagem, a identificação correta dos medicamentos e dispositivos associados à sua administração constitui um componente essencial da segurança do paciente na prática medicamentosa, promovendo uma prática centrada no cuidado do paciente. Assim a rotulagem deve ser realizada de forma imediata, sem interrupções, a fim de evitar erros de administração ou trocas indevidas^(21,29,34,35)^.

No que tange à administração de terapia medicamentosa intravenosa, evidenciou-se na literatura a realização da checagem da prescrição, verificação de alergias, informar o paciente sobre a medicação, confirmar sua identidade, além disso, observou-se, de forma recorrente na literatura analisada, a necessidade de avaliar o acesso vascular e verificar a sua permeabilidade^(23,26,31,35,37)^. A lavagem da linha de infusão entre diferentes medicamentos após a sua administração foi considerada uma técnica frequentemente utilizada como uma medida de segurança^(25-27,35,36)^. Também evidenciou-se a realização do registro da administração, da recusa do paciente e os eventos adversos ocorridos, bem como os sinais e sintomas observados, e as condutas realizadas^(35,37,38)^, como se pode ver a seguir (Figura 2).

**Figura 2:** Práticas seguras na terapia medicamentosa intravenosa, Rio das Ostras, Rio de Janeiro, Brasil, 2025

## DISCUSSÃO

A revisão de escopo identificou as práticas seguras na terapia medicamentosa intravenosa em pacientes adultos hospitalizados. Tais práticas contemplam o rigor técnico e a observância de protocolos assépticos em todas as etapas do processo, desde o preparo até o seu registro, assim, contribuindo diretamente para redução de riscos e auxiliando na prevenção de infecções relacionadas à assistência à saúde, promovendo a segurança do paciente.

A adoção de técnicas assépticas na pré-administração, administração e na pós-administração de terapia medicamentosa intravenosa é essencial para prevenção de infecções relacionados à assistência de saúde. Na presente revisão de escopo, destacou-se a higiene das mãos antes, durante e após a administração de terapia medicamentosa intravenosa - estudo transversal (E5); documento técnico da CDC (E12); documento técnico da OMS (E14). Esses achados da revisão de escopo corroboram com os resultados de um estudo realizado em um hospital de grande porte, em uma cidade no interior do Estado de São Paulo, nas clínicas médica e cirúrgica, em que, das 385 observações realizadas do processo de administração de medicamento endovenoso, em todas foi possível observar a higienização das mãos antes e após o preparo da medicação^(12)^, padrão que deve ser seguido de forma rotineira nas instituições de saúde.

A higiene das mãos é considerada uma prática segura na terapia medicamentosa intravenosa em pacientes adultos hospitalizados e sua baixa adesão é uma das principais causas associadas às infecções relacionadas à assistência à saúde (IRAS)^(39)^ e, apesar das recomendações de órgãos oficiais como MS e ANVISA - documentos técnicos (E13; E18), mesmo sendo uma técnica simples, de baixo custo e adequadas para prevenção e controle das IRAS, a higiene das mãos constitui-se um desafio da prática de enfermagem no contexto nacional e internacional. Um estudo realizado em um hospital privado na Bahia identificou a baixa adesão a higiene das mãos como um dos erros no preparo e na administração de medicamentos endovenosos, de igual maneira, um estudo internacional realizado na Etiópia observou que apenas 54 (24,0%) dos profissionais realizaram a higiene das mãos nos momentos necessários^(7,39,40)^.

As medidas assépticas, como a desinfecção do diafragma de frascos e gargalos de ampolas de medicações no preparo e a limpeza dos conectores do acesso venoso antes da administração - estudo transversal (E3); documento técnico (E11), são práticas seguras evidenciadas no escopo da literatura analisada e que também têm sido negligenciadas, contudo são essenciais para garantir a manutenção da técnica asséptica durante a terapia medicamentosa IV em adultos. Essas medidas, apesar de simples, previnem a propagação das IRAS, tendo em vista que negligenciar tal etapa pode acarretar a contaminação do sítio de infusão e da pele do paciente, aumentando, assim, o risco de flebite e infecção da corrente sanguínea. Esses achados vão ao encontro de um estudo realizado em um hospital referência em doenças infectocontagiosas em Fortaleza, que destacou que dentre as 111 observações realizadas do preparo e administração de medicamento, apenas 13 (11,7%) realizaram a desinfecção da ampola ou do frasco^(40,41)^.

Na etapa do preparo, destacou-se a necessidade da dupla checagem com a prescrição médica e verificação de todas as informações importantes - estudo prospectivo (E4); estudo observacional e prospectivo (E2), como práticas seguras na terapia medicamentosa intravenosa em pacientes adultos hospitalizados. Tais práticas quando negligenciadas pelo profissional podem acarretar em erros de medicação, como como foi identificado em um estudo realizado em um hospital no Estado da Bahia, Brasil, cuja taxa de erros no preparo das medicações intravenosas foi de 60% e um dos principais erros encontrados durante o preparo de medicações, foi a identificação incorreta da medicação preparada, desse modo nota-se a importância da dupla checagem para verificar todas as informações necessárias e garantir a segurança do paciente^(40)^. Vale ressaltar que as unidades hospitalares possuem papel fundamental na promoção da segurança do paciente ao incorporarem na instituição documentos ou protocolos com apresentação de informações acerca da diluição, reconstituição, concentração de medicamentos, como também o treinamento e capacitações dos profissionais de saúde^(42)^, reforçando, desse modo, a responsabilidade compartilhada entre instituição e profissionais na promoção da segurança do paciente.

Na etapa de rotulagem, a identificação correta e imediata dos medicamentos e dispositivos associados à sua administração - documentos técnicos (E11; E15); estudo observacional (E1); estudo experimental (E9), constituem-se como componentes essenciais para a segurança do paciente na terapia medicamentosa intravenosa em pacientes adultos hospitalizados, promovendo prática centrada no paciente. Destaca-se que a rotulagem deve conter as informações completas como medicamento, unidade, dose, tipo de diluente, volume total a ser administrado, horário e nome de quem preparou^(42-44)^. A identificação das medicações através da rotulagem após o momento do preparo, constitui uma barreira de segurança importante, antes da administração do medicamento. É uma estratégia de baixo custo, que organiza o processo de trabalho evitando que o paciente seja acometido por possíveis erros, como troca de medicações e sinalização de alergias medicamentosas, que podem acontecer em todas as fases da terapia medicamentosa intravenosa^(42-44)^.

A etapa de administração envolve diversos momentos essenciais que visam prevenir eventos adversos e garantir as práticas seguras na terapia medicamentosa intravenosa em pacientes adultos hospitalizados, que vão desde revisão da prescrição médica, identificação do paciente, verificação de possíveis alergias do paciente, informação sobre o que está sendo administrado até o esclarecimento de dúvidas - estudo qualitativo prospectivo (E4); estudo observacional (E6); estudo transversal (E8). Essas práticas seguras compiladas na presente revisão de escopo, apesar de simples e pautadas especialmente nas revisões, verificações e checagens, são frequentemente negligenciadas ou não realizadas, como pode ser observado em um estudo realizado em um hospital de referência do estado regional de Amhara, na Etiópia, que destacou que das 109 doses de medicamentos administradas, apenas em 11% houve práticas seguras. Os maiores erros observados nessa etapa foram: não esclarecer e/ou informar o que está sendo administrado ao paciente e não realizar a avaliação do paciente, após a administração do medicamento, momento essencial para identificação de possíveis eventos adversos^(45)^.

A identificação do paciente com pelo menos dois indicadores como nome completo e número do prontuário, também foi apontada em outro estudo como uma das principais barreiras, demostrando assim como na presente revisão, a necessidade da identificação correta do paciente no momento da administração do medicamento, garantindo a prática segura na terapia medicamentosa intravenosa em pacientes adultos hospitalizados. Um estudo realizado em um hospital no interior de São Paulo apontou que 79% dos pacientes entrevistados relataram que não houve qualquer tipo de conferência com seu nome antes da administração do medicamento. Essas evidências demostram a importância da adesão dessa boa prática para a segurança na assistência, pois contribuem para diminuição da taxa de danos evitáveis^(42,46)^. Desse modo, nota-se então que a adoção das práticas segura na terapia medicamentosa intravenosa em pacientes adultos hospitalizados, relacionadas a dupla checagem da prescrição no momento da administração, verificação da identificação do paciente e a necessidade de conhecimento de alergias dos pacientes contribuem para a redução de erros de medicamentos^(47)^.

Evidenciou-se como prática segura na terapia medicamentosa intravenosa em pacientes adultos hospitalizados, no escopo da literatura analisada, a necessidade de realização da avaliação do local do acesso vascular e a verificação da permeabilidade antes da administração da terapia medicamentosa intravenosa, como também a lavagem da linha de infusão após administração de cada medicação - estudo transversal (E7); estudo misto (E10); parecer técnico (E17). Assim, a realização da técnica de *flushing*, lavagem da linha de infusão antes, entre e após as medicações é um cuidado essencial da equipe de enfermagem, que visa avaliar e manter a permeabilidade do acesso vascular, prevenindo a sua obstrução, contribuindo para a segurança do paciente. A literatura analisada destaca que o *flushing* deve ser realizado com soluções fisiológica 0,9% e, apesar da sua recomendação em diretrizes e *guidelines*, a prática ainda é permeada pela baixa adesão como foi identificado em um estudo realizado em um hospital no interior de São Paulo, Brasil, em que somente 4,81% das administrações tiveram a realização tal técnica^(12,48)^. Tais discrepâncias entre o que é preconizado e o que é efetivamente praticado suscitam discussões sobre as barreiras que dificultam a incorporação de práticas seguras no cotidiano assistencial e a necessidade de estratégias educativas e institucionais para sua consolidação.

A etapa do registro constitui-se como uma prática segura que deve ser realizada na terapia medicamentosa intravenosa em pacientes adultos hospitalizados, de acordo com o escopo da literatura, uma vez que o registro da recusa de medicações, intercorrências, eventos adversos e a checagem da medicação administrada contribuem para a rastreabilidade do cuidado, a continuidade terapêutica e a prevenção de erros relacionados à administração de medicamentos - livro (E16); protocolo (E18). Corroborando com as práticas seguras encontradas na presente revisão de escopo, um estudo realizado em um hospital da cidade de Fortaleza no Brasil, apontou que 92 (82,8%) dos profissionais de enfermagem registram e checam após administração, no entanto apenas 20 (18%) registraram as queixas e as reações adversas apresentadas pelo paciente^(41,47)^, demonstrando que apesar de realizarem uma prática segura, os profissionais ainda não a realizam de forma completa.

As práticas seguras evidenciadas nesta revisão de escopo fazem parte de um conjunto de medidas que são necessárias em toda administração de terapia medicamentosa IV, contudo há que se considerar as particularidades em cada fase da vida dos pacientes. Um estudo realizado sobre a temática em pacientes pediátricos, apresenta particularidades importantes a serem consideradas para este público específico, que não foi a população escolhida para a presente revisão, como a imaturidade fisiológica, a indisponibilidade de formulações medicamentosas próprias para a faixa etária e as subdoses que precisam ser administradas e que estão intimamente relacionadas ao peso e idade do paciente^(49)^.

No contexto nacional, observa-se que os profissionais de enfermagem enfrentam diversas barreiras para a realização de práticas seguras na terapia medicamentosa intravenosa em pacientes adultos hospitalizados, tais como sobrecarga de trabalho, falhas na comunicação entre equipes, escassez de recursos tecnológicos e ausência de uma cultura institucional voltada à segurança, fatores que contribuem para a ocorrência de práticas inadequadas e potencialmente inseguras^(50)^. Em âmbito internacional, essas dificuldades também são amplamente reconhecidas e têm sido abordadas pela OMS, que inclui, entre suas metas globais de segurança do paciente, a administração segura de medicamentos^(50)^. Diversos países vêm implementando tais diretrizes e investindo em tecnologias de apoio, como sistemas informatizados de prescrição e identificação eletrônica do paciente, além de promoverem uma cultura organizacional baseada na notificação de incidentes e na educação permanente^(50)^. Desse modo, embora os contextos institucionais e socioculturais variem, evidencia-se a necessidade universal de fortalecer a implementação de práticas seguras na administração da terapia medicamentosa intravenosa^(50)^. Assim, mapear as evidências científicas fornece um panorama atual que pode contribuir para diminuir uma lacuna voltada à prática da enfermagem e ao cuidado seguro na terapia medicamentosa intravenosa em adultos.

### Limitações do estudo

A presente revisão de escopo apresenta limitações, como a ausência de descritores específicos para a temática o que pode ter levado a limitar a amostra e a não inclusão de estudos relevantes. Além disso, destaca-se que alguns artigos não foram incluídos por não estarem disponíveis na íntegra.

Não foi realizada a avalição da qualidade metodológica dos estudos incluídos, dessa forma, as práticas identificadas como seguras foram descritas com base nas recomendações dos próprios estudos, sem realização da análise do nível de evidência. A heterogeneidade dos estudos incluídos na revisão, quanto aos contextos institucionais, profissionais envolvidos, tipos de medicamentos e tecnologias utilizadas limitam a generalização dos achados.

### Contribuições para a Área

Este estudo oferece contribuições significativas para prática de enfermagem ao evidenciar a importância da adoção das práticas seguras na terapia medicamentosa intravenosa no contexto hospitalar. Ao realizar o mapeamento de práticas que promovem a segurança do paciente, o estudo reforça o papel estratégico da equipe de enfermagem na prevenção de erros de medicação. As evidências encontradas contribuem não apenas para fortalecer a qualidade da assistência prestada, mas também como subsídio valioso para formação, gestão e prática profissional em enfermagem.

## CONCLUSÕES

Este estudo contribui para o mapeamento das evidências científicas sobre práticas seguras na terapia medicamentosa intravenosa em pacientes adultos hospitalizados, oferecendo subsídios relevantes para a melhoria da segurança do paciente. As principais medidas identificadas incluem a utilização de técnicas assépticas, a dupla checagem das prescrições, a rotulagem padronizada dos medicamentos e o registro sistemático da administração e de possíveis reações adversas. Essas ações representam etapas críticas do processo medicamentoso e são determinantes para a qualidade e a segurança do cuidado.

Os achados reforçam a necessidade de capacitação contínua das equipes de enfermagem, adoção de protocolos institucionais e fortalecimento da cultura de segurança no contexto hospitalar. Evidenciou-se a importância da implementação de políticas institucionais e normativas que padronizem as práticas seguras, promovam auditorias sistemáticas e assegurem condições de trabalho adequadas para o exercício seguro da administração medicamentosa. Assim, este estudo subsidia tanto a prática clínica quanto a formulação de políticas voltadas à segurança do paciente e à qualidade assistencial.

## Data Availability

Os dados de pesquisa estão disponíveis em repositório: https://osf.io/hv7g2.
